# A Better Understanding of Atrial-like and Ventricular-like Action Potentials in Stem Cell-Derived Cardiomyocytes: The Underestimated Role of the L-Type Ca^2+^ Current

**DOI:** 10.3390/cells14161226

**Published:** 2025-08-08

**Authors:** Arie O. Verkerk, Christiaan C. Veerman, Maaike Hoekstra, Harsha D. Devalla, Ronald Wilders

**Affiliations:** 1 Department of Medical Biology, Amsterdam Cardiovascular Sciences, Amsterdam University Medical Center, University of Amsterdam, 1105 AZ Amsterdam, The Netherlands; a.o.verkerk@amsterdamumc.nl (A.O.V.); h.d.devalla@amsterdamumc.nl (H.D.D.); 2 Department of Experimental Cardiology, Heart Center, Amsterdam Cardiovascular Sciences, Amsterdam University Medical Center, University of Amsterdam, 1105 AZ Amsterdam, The Netherlands; c.c.veerman@amsterdamumc.nl (C.C.V.); m.hoekstra@hagaziekenhuis.nl (M.H.)

**Keywords:** stem cell-derived cardiomyocytes, patch clamp, action potential, transient outward K^+^ current, delayed rectifier K^+^ current, L-type Ca^2+^ current, Na^+^-Ca^2+^ exchange current, Ca^2+^ transient, SERCA, intracellular Ca^2+^ homeostasis

## Abstract

Human embryonic stem cell-derived cardiomyocytes (hESC-CMs) tend to show a mixed population of action potential (AP) types, including atrial-like (A-like) and ventricular-like (V-like) APs. In the present study, we investigated the membrane currents underlying these two AP types in hESC-CMs. These were generated using standard (Std) and retinoic acid (RA)-based differentiation protocols. Patch clamp methodology was used to correlate AP morphology with major cardiac ion currents by applying alternating current and voltage clamp protocols to each cell, and to measure L-type Ca^2+^ current (I_Ca,L_) and Na^+^-Ca^2+^ exchange current (I_NCX_) in detail, whereas Ca^2+^ transients were measured ratiometrically using Indo-1. A- and V-like APs were found in both Std and RA-treated hESC-CMs and the AP plateau amplitude (AP_plat_), as a measure of fast phase-1 repolarization, appeared the best AP criterion to separate these two AP types. Traditional voltage clamp experiments revealed a significantly smaller I_Ca,L_ density in RA-treated hESC-CMs, as well as larger densities of the transient outward and delayed rectifier K^+^ currents (I_to1_ and I_K_, respectively), without changes in the inward rectifier K^+^ current (I_K1_). The AP_plat_ showed strong and moderate correlations with the densities of I_Ca,L_ and I_K_, respectively, in the absence of a clear-cut correlation with the density of I_to1_. Using pre-recorded, typical A- and V-like APs, AP clamp demonstrated that the I_Ca,L_-mediated Ca^2+^ influx during the V-like AP in Std hESC-CMs is 3.15 times larger than the influx during the A-like AP in RA-treated hESC-CMs. Ca^2+^ transients of A-like hESC-CMs have a lower diastolic and systolic level, as well as a lower amplitude, than those of Std hESC-CMs, while their duration is shorter due to enhanced SERCA activity. In conclusion, I_Ca,L_ is an important determinant of the differently shaped A- and V-like APs in hESC-CMs. Furthermore, the Ca^2+^ homeostasis differs between A- and V-like hESC-CMs due to the smaller I_Ca,L_ and enhanced SERCA activity during A-like APs, resulting in a strongly reduced Ca^2+^ influx, which will cause a substantial reduction in I_NCX_, further contributing to the shorter A-like APs.

## 1. Introduction

Human embryonic stem cell-derived cardiomyocytes (hESC-CMs) and human induced pluripotent stem cell-derived cardiomyocytes (hiPSC-CMs) have become one of the most popular model systems for cardiovascular research over the last decade [1,2,3,4,5]. This reflects the difficulty of obtaining freshly isolated human cardiomyocytes, in combination with a high degree of species dependence of cardiac electrophysiology, including action potential (AP) duration (APD) and shape, that limits the applicability of animal models [6,7]. Further advantages of hESC-CMs and hiPSC-CMs, in addition to their relative ease of availability, is that they can be generated from patient tissue and that they can be genetically modified, making them extremely useful for studying genes and transcription factors underlying arrhythmias [8] and cardiomyopathies [9,10] as well as development [11]. Also, acute and long-term drug studies are relatively easy to perform due to the ability to keep hESC-CMs and hiPSC-CMs in prolonged culture [12]. Finally, (dys)function can be studied not only with very precise methods, but also with rapid screening and relatively easy to perform techniques [13,14,15,16,17,18], enabling personalized disease modeling and drug testing. On the other hand, the wide variability in experimental approaches thus far—including variability across stem cell lines and differentiation protocols—among studies of stem cell-derived cardiomyocytes has led to limited reproducibility and uncertainty in the interpretation of the obtained data [19,20,21].

Huge efforts have been invested in the development of methods for reprogramming, cell differentiation, and generation of engineered heart tissue systems [22,23,24]. However, standard (Std) differentiation protocols, i.e., protocols not specifically designed to push cells toward an atrial or nodal phenotype, tend to result in heterogeneous populations of hiPSC-CMs and hESC-CMs (Table 1) that are typically categorized as nodal-like (N-like), atrial-like (A-like), or ventricular-like (V-like), while Streckfuss-Bömeke et al. [25] also described Purkinje-like (P-like) hiPSC-CMs. Many studies, but not all, categorized the hiPSC-CMs or hESC-CMs of interest into AP shapes according to specific and well-defined AP criteria, as explained in detail below and summarized in Table 1.

Table 1 clearly shows that the AP criteria used to define hiPSC-CMs and hESC-CMs as N-like, A-like, or V-like vary widely between studies, with little standardization to date. A number of studies used the absolute APD and longer APDs at 30, 50, or 90% of repolarization (APD_30_, APD_50_, and APD_90_, respectively) to define APs as V-like. Jara-Avaca et al. [36] also included the ratio of APD_90_ to APD_50_ (APD_90/50_), which was used as a measure for a triangular AP shape, and defined APs with a APD_90/50_ < 1.4 as V-like. Apart from the absolute APD values, Bett et al. [37] and Kim et al. [38] also included a measure of triangulation, but used the ratio of APD_30_ to APD_90_ (APD_30/90_). Many other studies have used a measure of triangulation as the sole parameter to distinguish between A- and V-like APs. This measure was either one of the already mentioned ratios or the one introduced by Ma et al. [44], who measured the time difference between APD_30_ and APD_40_ (APD_30–40_) and the time difference between APD_70_ and APD_80_ (APD_70–80_), and categorized APs of hiPSC-CMs differentiated with a Std protocol into V-like and A-like with an APD_30–40_/APD_70–80_ ratio > 1.5 and <1.5, respectively.

Although the aforementioned quantitative AP criteria for distinguishing between different AP shapes of hiPSC-CMs and hESC-CMs may be correct at least to some extent, there is quite some debate about their usefulness because of the numerous factors that affect AP parameters [50,51,52,53,54]. To start, the absolute APD value used to separate into A- and V-like cells is highly sensitive to experimental settings, such as cell line, culture media, and differentiation method [55,56,57]. Recording conditions, including temperature and the amount of EGTA, if any, in the recording pipette also have a huge impact on APD. Furthermore, APD analysis is very sensitive to the acquired AP amplitude and the associated peak membrane potential [58], so that the APD, and in particular the APD_20_, in cells with a reduced APA will be analyzed in a different voltage range than in cells with a high APA and thus is a measure of the activity of a different set of ion currents [59,60]. Therefore, we introduced an AP parameter that is independent of APDs, i.e., the voltage amplitude of the AP plateau (AP_plat_) phase at 20 ms after the AP upstroke, to distinguish between A-like and V-like APs of hESC-CMs and hiPSC-CMs [49,61,62]. This AP_plat_ is a measure of repolarization during the early AP phase, and using a retinoic acid (RA)-based differentiation protocol we pushed hESC-CMs toward a more transcriptional and electrophysiological atrial phenotype with enhanced expression of *KCNA5* and its associated ultrarapid delayed rectifier K^+^ current (I_Kur_) [49], and defined APs with an AP_plat_ < 80 mV as A-like, while those with an AP_plat_ > 80 mV as V-like. The hESC-CMs with an A-like AP were sensitive to various atrial-specific drugs [49], with for example an increased AP_plat_ upon I_Kur_ blockade, and the low AP_plat_ agrees with that of freshly isolated human atrial cardiomyocytes [60,63].

Table 1 (rightmost column) also summarizes the percentage of each of the three commonly described cell subtypes (i.e., N-like, A-like, and V-like) obtained with the specific criteria used in each of the studies referred to (including the limitations of these specific criteria, as set out above), demonstrating that these percentages vary widely between studies. Factors that may contribute to the relative abundance of a particular AP shape may include the cell line used, the differentiation protocol, and likely also the degree of maturation, although the latter gives conflicting results. Zhang et al. [47] reported an increased percentage of cells displaying V-like APs between 2 and 4 weeks. However, this is in contrast to the findings of Sheng et al. [64], who found that the percentage of hiPSC-CMs with V-like APs decreased from 79% (day 20) to 49% (day 60), while Seibertz et al. [65] reported unchanged proportions of cardiac subtypes throughout hiPSC-CM culture. Sheng et al. [64] found that the relative abundance of AP shapes in hESC-CMs was also almost unchanged between day 20 and day 60. Interestingly, hiPSC-CMs at day 60 displayed three major shapes of APs with only a slight excess of V-like as compared to A-like AP shapes (V-like 49%, A-like 39%, N-like 12%), in contrast to hESC-CMs at day 60, where the majority of cells revealed V-like APs (V-like 80%, A-like 18%, N-like 2%).

Studies of cardiac cell subtype-related diseases, heart chamber-specific drugs, and development require pure populations of a desired cell type [66] and/or detailed phenotyping of N-, A-, and V-like hiPSC-CMs [67]. However, how a specific N-, A-, or V-like AP morphology is linked to major ion current densities has been studied only to a limited extent. In general, voltage clamp experiments are performed in mixed cell-type populations and without recording of APs to identify whether a cardiomyocyte is N-, A-, or V-like [44]. Only Lieu et al. [68] defined A- or V-like hiPSC-CMs based on AP measurements and subsequently performed voltage clamp experiments to determine differences in hyperpolarization-activated current (I_f_), L-type Ca^2+^ current (I_Ca,L_), and rapid delayed rectifier K^+^ current (I_Kr_). A-like hiPSC-CMs had a smaller depolarizing inward I_Ca,L_, but also a smaller repolarizing outward I_Kr_, complicating a direct explanation for the A-like APs.

The contribution of a particular subtype to the obtained cell population can be increased by specific differentiation protocols, as shown in Table 1. This has provided crucial data on electrophysiology and intracellular Ca^2+^ homeostasis for hESC-CMs and/or hiPSC-CMs generated with Std [44] and more specific differentiation protocols for N-like [69,70,71,72,73] and A-like [45,49,74,75,76,77] hESC-CMs and hiPSC-CMs, but there are still mixed populations of cell types (see Table 1), which hampers detailed phenotyping [74]. For example, Altomare et al. [39] recently tested the effects of 50 µmol/L 4-aminopyridine (4-AP) to block I_Kur_ of hiPSC-CMs with A-like and V-like APs that were categorized as such based on their APD_20/90_ ratio (Table 1). Approximately 20% of the A-like APs were insensitive to 4-AP, thus indicating the absence of the atrial-specific I_Kur_ in these cells. Conversely, V-like APs did not respond to 4-AP in around 80% of the cells, while in around 20% an AP prolongation was found. This indicates that even those hiPSC-CMs categorized as A-like or V-like do not necessarily have an ionic phenotype that belongs to the subtype based on AP shape.

In the present study, we focused on hESC-CMs, carrying out an in-depth characterization of their A- and V-like APs in relation to the underlying individual ion currents. Therefore, we followed the approach of Lieu et al. [68] and correlated cardiac phenotype based on AP morphology and major cardiac ion currents by applying both current clamp and voltage clamp protocols to each cell. Thus, we first measured APs of a cell and then carried out voltage clamp experiments on the same cell. We found a strong relationship between I_Ca,L_ and AP_plat_, and in subsequent experiments, we studied I_Ca,L_, the Na^+^-Ca^2+^ exchange (NCX) current (I_NCX_), and Ca^2+^ transients of Std and RA-differentiated hESC-CMs in more detail. The AP-clamp technique was further used to specify the role of the AP shape in Ca^2+^ influx through I_Ca,L_. We used a Std differentiation protocol as well as an RA-based differentiation protocol [78] to increase the amount of A-like APs in the hESC-CMs [49].

## 2. Materials and Methods

### 2.1. hESC Maintenance, Differentiation, and Dissociation

#### 2.1.1. hESC Maintenance and Differentiation to Cardiomyocytes

No animal experiments have been performed in the present study. hESC-CMs were generated as described previously in detail [49]. In short, undifferentiated colonies of *NKX2-5^eGFP/w^* hESCs [79], with Cellosaurus ID HES-3 NKX2.5eGFP/w (CVCL_A8JS) [80], were maintained on irradiated mouse embryonic fibroblasts and differentiation (here defined as Std differentiation) to cardiomyocytes was performed using a Spin-Embryoid Body (Spin-EB) protocol [49]. Therefore, hESCs were harvested and resuspended on day 0 in BPEL (Bovine Serum Albumin (BSA) Polyvinylalcohol Essential Lipids) medium [81] supplemented with 20–30 ng/mL hActivin-A (R&D Systems, Minneapolis, MN, USA), 20–30 ng/mL bone morphogenetic protein 4 (R&D Systems), 40 ng/mL stem cell factor (STEMCELL Technologies, Vancouver, BC, Canada), 30 ng/mL vascular endothelial growth factor (R&D Systems) and 1.5 μmol/L CHIR 99021 (Axon Medchem, Groningen, The Netherlands). EBs were refreshed on day 3 with BPEL and then transferred to gelatin-coated dishes on day 7. To increase the number of hESC-CMs with an atrial-like identity [49], 1 μmol/L all-*trans*-retinoic acid (RA) (Sigma, Burlington, MA, USA) was added from day 4 to 7 of differentiation. Following plating at day 7, BPEL was refreshed every 3–4 days.

#### 2.1.2. Single Cell Preparation

Spin-EBs resulting from Std and RA-treated differentiations were dissociated at day 14 to obtain single cells using TrypLE (Life Technologies, Carlsbad, CA, USA). Single cells were plated on gelatin-coated coverslips and electrophysiological measurements were performed after 7–10 days. Coverslips containing the cells were put into a temperature-controlled (36 ± 0.1 °C) recording chamber [82] mounted on the stage of an inverted microscope (Nikon Diaphot, Nikon, Tokyo, Japan), and superfused with modified Tyrode’s solution containing (in mmol/L): NaCl 140, KCl 5.4, CaCl_2_ 1.8, MgCl_2_ 1.0, glucose 5.5, HEPES 5.0; pH 7.4 (NaOH). We selected single GFP^+^ myocytes that were spontaneously beating or that were intrinsically quiescent but able to contract upon field stimulation [83].

### 2.2. Patch Clamp Experiments

#### 2.2.1. Data Acquisition

APs and membrane currents were recorded using an Axopatch 200B amplifier (Molecular Devices, Sunnyvale, CA, USA). Voltage control and data acquisition were realized with custom software (‘Scope’, version 04.04.27). Data analysis was also performed with custom software (‘MacDaq’, version 8.0). Signals were low-pass filtered with a cut-off frequency of 5 kHz and digitized at 5 kHz for spontaneous APs, 40 kHz for stimulated APs, 4 kHz for net membrane currents, 10 kHz for I_Ca,L_, and 1 kHz for I_NCX_. Cell membrane capacitance (C_m_, in pF) was estimated as described in detail elsewhere [84]. Patch pipettes were pulled from borosilicate glass (Harvard Apparatus, Waterbeach, UK) using a custom vertical microelectrode puller and had a resistance of 2–3 MΩ after filling with the pipette solutions as indicated below. All potentials were corrected for the estimated liquid junction potential [85]. C_m_ and series resistance were compensated for at least 80% for I_Ca,L_ and I_NCX_, and for ≈60% for net membrane currents. For all cell types and experiments, data were collected from at least 3 independent differentiations.

#### 2.2.2. Action Potentials

APs were measured by the amphotericin-perforated patch clamp methodology. The external bath solution was modified Tyrode’s solution; pipettes were filled with a solution containing (in mmol/L): K-gluconate 125, KCl 20, NaCl 5, amphotericin-B 0.44, HEPES 10, pH 7.2 (KOH). We recorded spontaneous APs or APs elicited at 1 Hz by 3-ms, 1.2–1.4× threshold current pulses through the patch pipette in quiescent cells (see Section 3.1.1 below). We analyzed cycle length, maximum diastolic potential (MDP), AP amplitude (APA), AP plateau amplitude at 20 ms after the AP upstroke (AP_plat_), and AP duration at 20, 50, and 90% repolarization (APD_20_, APD_50_, and APD_90_, respectively). Parameters from 10 consecutive APs were averaged.

#### 2.2.3. Membrane Currents

Net membrane currents were measured by the amphotericin-perforated patch clamp technique, and the pipette and bath solutions were as described in Section 2.2.2. The inward rectifier K^+^ current (I_K1_), the delayed rectifier K^+^ current (I_K_), and I_Ca,L_ were examined by 500-ms voltage clamp steps every 2 s to membrane potentials ranging from −100 to +50 mV. The holding potential was set at −50 mV to inactivate the Na^+^ current (I_Na_) and the transient outward K^+^ current (I_to1_). I_Ca,L_ was analyzed as the current difference between the peak and quasi-steady-state current at the end of the voltage clamp step. I_K1_ and I_K_ were defined as the quasi-steady-state current at the end of the voltage clamp steps at potentials negative or positive to −50 mV, respectively. I_to1_ was examined by 1 s voltage clamp steps every 10 s from a holding potential of −80 mV to membrane potentials ranging from −80 to +40 mV. The voltage dependence of inactivation was determined by a two-step voltage clamp protocol from a holding potential of −80 mV. The first, 1 s, step was to potentials ranging from −80 to +40 mV. The second, 500 ms, step was to +40 mV. I_to1_ was measured in the presence of 0.25 mmol/L CdCl_2_, which blocks I_Ca,L_ [86] and strongly inhibits I_Na_ [87]. Suppression of these inward currents allows a more accurate determination of I_to1_. Each of these voltage clamp protocols are shown graphically in Section 3.1.2 near the associated membrane current recordings.

Detailed I_Ca,L_ and I_NCX_ measurements were performed by the ruptured patch clamp methodology with strongly modified pipette and bath solutions and specific protocols. I_Ca,L_ was measured with a two-pulse square-step voltage clamp protocol with a cycle length of 2 s. The bath solution contained (in mmol/L): TEA-Cl 145, CsCl 5.4, CaCl_2_ 1.8, MgCl_2_ 1.0, HEPES 5.0, pH 7.4 (NMDG-OH). Pipettes were filled with a solution containing (in mmol/L): CsCl 145, HEPES 10, EGTA 10, K_2_ATP 5, pH 7.2 (NMDG-OH). I_Ca,L_ was defined as the difference between the peak current and the steady-state current. In a subset of cells, I_Ca,L_ was measured during AP clamp measurements as nifedipine-sensitive current. Therefore, we used a voltage clamp protocol constructed from pre-recorded APs of a typical Std and a typical RA-treated hESC-CM and washed in 5 µmol/L nifedipine, which completely blocks I_Ca,L_ in stem cells [88]. I_NCX_ was measured as 10 mmol/L NiCl_2_-sensitive current during a descending voltage ramp protocol [89]. The pipette solution contained (mmol/L): CsCl 145, NaCl 5, Mg-ATP 10, TEA 10, HEPES 10, EGTA 20, CaCl_2_ 10 (calculated free Ca^2+^: 150 nmol/L); pH 7.2 (NMDG-OH). To suppress membrane currents other than I_NCX_, the following blockers were added to a K^+^-free Tyrode’s solution (in mmol/L): BaCl_2_ 1, CsCl 2, nifedipine 0.005, ouabain 0.1, DIDS 0.2 [90]. Each of these voltage clamp protocols are shown graphically in Section 3.2 near the associated membrane current recordings.

Current densities were calculated by dividing current amplitudes by C_m_. Steady-state activation and inactivation curves were fitted using a Boltzmann equation:

I/I_max_ = A/{1.0 + exp[(V_1/2_ − V)/k]}, (1)

in which V_1/2_ is the half-maximum (in)activation potential and k is the slope factor. The decay of I_CaL_ was fitted with the double exponential equation:

I/I_max_ = A_f_ × exp(−t/τ_f_) + A_s_ × exp(−t/τ_s_), (2)

where A_f_ and A_s_ are the fractional amplitudes, and τ_f_ and τ_s_ the time constants of the fast and slow components, respectively.

### 2.3. Intracellular Ca^2+^ Measurements

Single cells were prepared for Ca^2+^ measurements as described in Section 2.1, with the exception that we used 2.4 mmol CaCl_2_ in the modified Tyrode’s solution (37 °C). Intracellular Ca^2+^ transients were measured at 1 Hz, using the fluorescent probe Indo-1, as previously described in detail [91]. In short, the intracellular Ca^2+^ concentration ([Ca^2+^]_i_) was calculated using the formula:

[Ca^2+^]_i_ = β × K_d_ × (R − R_min_)/(R_max_ − R), (3)

where β is the ratio of maximum to minimum I505 (2.2) and K_d_ is the dissociation constant for Indo-1 AM, which is 250 nmol/L at 37 °C (data sheet for Indo-1 AM, Thermo Fisher Scientific, Waltham, MA, USA). R_min_ and R_max_ are the ratios at minimum and maximum [Ca^2+^]_i_, respectively. R_max_ was determined by blocking the Na^+^/K^+^ exchanger with gramicidin (2 μmol/L, Sigma-Aldrich, St. Louis, MO, USA), which rapidly causes [Ca^2+^]_i_ overload. R_min_ was analyzed in Ca^2+^-free modified Tyrode’s solution. In our hESC-CMs, R_min_ was 1.4 ± 0.09 (mean ± SEM, *n* = 12), and R_max_ was 5.1 ± 0.19 (*n* = 11).

We analyzed diastolic and systolic [Ca^2+^]_i_ concentrations, [Ca^2+^]_i_ transient amplitudes, and the time constant (τ) of the [Ca^2+^]_i_ transient decay. The decay rates of both systolic and caffeine-induced [Ca^2+^]_i_ transients were obtained by fitting single exponential functions to the decay phase of the transients. The amplitude of [Ca^2+^]_i_ transients evoked by application of 10 mmol/L caffeine and 10 mmol/L NiCl_2_ was taken as a measure of sarcoplasmic reticulum (SR) Ca^2+^ content [92]. The various mechanisms involved in Ca^2+^ extrusion from the cytoplasm were studied as we previously described in detail [91]. In short, the SR-dependent rate of Ca^2+^ uptake (SERCA activity; K_SERCA_) was calculated by subtracting the decay rate constant of the caffeine (10 mmol/L)-evoked Ca^2+^ transient (K_caff_) from the systolic Ca^2+^ transient decay rate constant (K_sys_). The contribution of NCX to Ca^2+^ removal from the cytoplasm was derived by subtracting the decay of the caffeine-induced Ca^2+^ transient (K_caff_) from the decay of the caffeine-induced Ca^2+^ transient in the presence of 10 mmol/L NiCl_2_ (K_Caff+Ni_) to block the NCX. The decay rate constants of the caffeine-induced Ca^2+^ transients in the presence of 10 mmol/L NiCl_2_ were used to assess the activity of the slow mechanisms (mitochondrial Ca^2+^ uptake and sarcolemmal Ca-ATPase) during [Ca^2+^]_i_ decay.

### 2.4. Statistics

Data are presented as mean ± SEM or box plots (with median and interquartile range). Statistical analysis was carried out with SigmaStat 3.5 (Systat Inc., St. Louis, MO, USA). Normality and equal variance assumptions were tested with the Kolmogorov–Smirnov and Levene median tests, respectively. Two groups were compared using the unpaired *t*-test or, if normality and/or equal variance tests failed, the Mann–Whitney rank-sum test. Three or more groups were compared by two-way ANOVA or two-way repeated measures (RM) ANOVA, followed by the Students–Newman–Keuls post hoc test. *p* < 0.05 was considered statistically significant.

## 3. Results

### 3.1. Action Potential Waveforms Explained by Ionic Currents

In the present section, we want to explain the existence of different AP morphologies of hESC-CMs by membrane current differences. To relate AP morphologies to individual membrane currents, we performed both current clamp and voltage clamp experiments on the same group of cells. We first measured APs (using current clamp) in a large group of cells, as described below in Section 3.1.1, and then performed voltage clamp experiments on a subset of these cells to characterize their major cardiac ion currents, as described below in Section 3.1.2. To obtain hESC-CMs, we used a Std differentiation protocol as well as an RA-based differentiation protocol to increase the amount of A-like APs [49]. The membrane current densities were plotted against the AP_plat_, which provides full insight into how the differences in AP shape are related to these currents (see Section 3.1.3).

#### 3.1.1. AP Parameters in Std and RA-Treated hESC-CM Populations

In a population of stem cell-derived cardiomyocytes, including hESC-CMs, a number of cells typically show spontaneous beating, while others are quiescent. We recorded APs from spontaneously beating hESC-CMs as well as from intrinsically quiescent hESC-CMs that were able to contract upon field stimulation. In the latter cells, APs were elicited at 1 Hz by current pulses through the patch pipette. Table 2 summarizes the average AP parameters of both the spontaneous and the stimulated hESC-CMs from Std and RA-based differentiations.

RA-treated hESC-CMs have a higher spontaneous firing rate than Std hESC-CMs, as indicated by their shorter cycle length. In addition, RA-treated hESC-CMs have shorter APD_20_ and APD_50_ in both spontaneous and stimulated APs, while their APD_90_ is only shorter during spontaneous activity (*p* = 0.72 in case of the stimulated APs). Furthermore, the APA and the AP_plat_ are significantly lower in RA-treated hESC-CMs in both spontaneous and stimulated APs. There are also clear differences between the spontaneous and the stimulated APs. Importantly, the MDP is more hyperpolarized in the stimulated cells in both Std and RA-treated hESC-CMs. However, the APA did not appear to be significantly increased (*p* = 0.14 (Std); *p* = 0.51 (RA-treated)). As a result of the more hyperpolarized MDP, the observed maximum AP upstroke velocity (dV/dt_max_) is substantially higher in the stimulated cells, due to a higher availability of Na^+^ channels at more negative membrane potentials [84,93]. The experimental data may suggest that the dV/dt_max_ of the intrinsically quiescent hESC-CMs is higher in Std than in RA-treated ones, but this increase did not reach the level of significance (*p* = 0.14). In addition, the APD values appear to be lower in the stimulated cells, which is most evident for the Std hESC-CMs.

**Figure 1:**
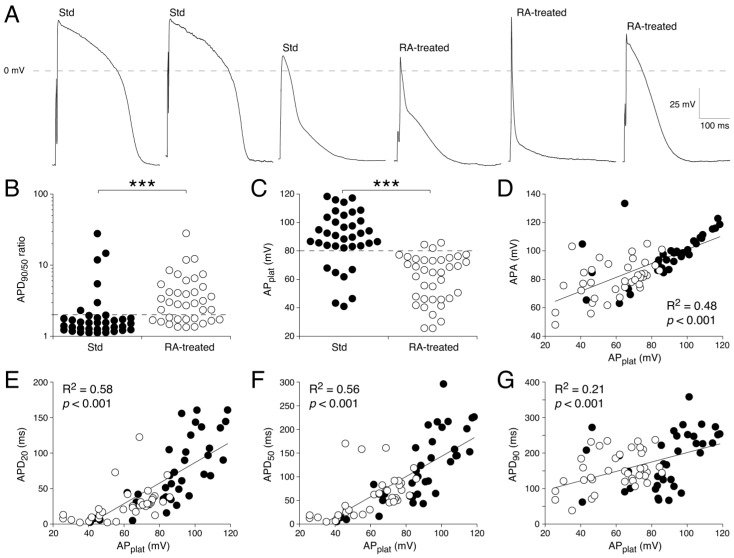
Action potentials (APs) of Std and RA-treated intrinsically quiescent hESC-CMs stimulated at 1 Hz, measured with amphotericin-perforated patch clamp technique. (**A**) APs of a selection of Std and RA-treated hESC-CMs, highlighting variability in shape. (**B**) Dot plot of APD_90/50_ ratio in Std and RA-treated hESC-CMs (*n* = 36 and *n* = 37, respectively). Dashed line marks APD_90/50_ ratio of 2. Note the logarithmic scale. (**C**) Dot plot of associated AP_plat_ values. Dashed line marks AP_plat_ of 80 mV. (**D**–**G**) Relationships between AP_plat_ and (**D**) APA, (**E**) APD_20_, (**F**) APD_50_, and (**G**) APD_90_. Closed and open symbols are Std and RA-treated cells, respectively. *** *p* < 0.001, RA-treated vs. Std (Mann–Whitney rank-sum test). Solid lines: linear correlation fits (Pearson correlation tests), with R^2^ and *p* values as indicated.

While these AP differences between Std and RA-treated hESC-CMs are consistent with many studies that have used RA to increase the number of cells with an atrial fate [31,34,45,48,61,62,72,74,75,76,77,94,95,96,97,98,99], there is a substantial heterogeneity in AP shape in both the Std and RA-treated hESC-CM populations, as illustrated in Figure 1A with stimulated APs. Of note, we restricted Figure 1 to the intrinsically quiescent cells stimulated at 1 Hz to rule out the possibility that the observed differences in AP parameters between the Std and the RA-treated cells are due to their differences in cycle length. To explore this heterogeneity in more detail, we generated dot plots of both the APD_90/50_ ratio and the AP_plat_ of the stimulated cells, as shown in Figure 1B,C, respectively. Both dot plots highlight the heterogeneity, but the AP_plat_ seems to be a sharper discriminating factor to separate V- and A-like APs, especially in the RA-treated group. Using an APD_90/50_ < 2, the APD_90/50_ ratio defined 83% of the Std cells (*n* = 36) as V-like (Figure 1B, dashed line). According to this ratio, 35% of the RA-treated hESC-CMs (*n* = 37) would be defined as V-like (Figure 1B, right). Using the AP_plat_ with a cut-off value of 80 mV (Figure 1C, dashed line) [49], 81% of the Std cells would have V-like APs (Figure 1C, left). These are exactly the same cells as found using the discriminating APD_90/50_ < 2, except for one cell that was V-like with the APD_90/50_ < 2 but did not have an AP_plat_ > 80 mV. However, using the AP_plat_ with a cut-off value of 80 mV, just 8% of the RA-treated cells would be defined as V-like (Figure 1C, right), which is thus substantially lower than the 35% obtained using the APD_90/50_ approach.

We next determined the linear correlation between the AP_plat_ and each of the other repolarization-related AP parameters, i.e., APA, APD_20_, APD_50_, and APD_90_ (Table 2). Figure 1E,F, shows that AP_plat_ is strongly correlated with both APD_20_ and APD_50_ (as indicated by their high R^2^). This is consistent with the idea that AP_plat_ is a strong parameter for the repolarization rate of the early AP phase, which is substantially larger in stem cell-derived cardiomyocytes with an atrial fate [100]. The correlation of AP_plat_ with APA is also strong (Figure 1D), but its correlation with APD_90_ is only moderate (Figure 1G).

#### 3.1.2. Net Membrane Currents in Std and RA-Treated hESC-CM Populations

**Figure 2:**
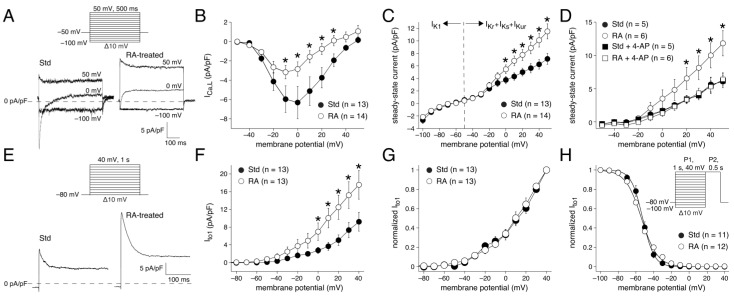
Membrane currents in intrinsically quiescent Std and RA-treated hESC-CMs. (**A**) Voltage clamp protocol (top panel) and typical currents recorded at −100, 0, and +50 mV in a Std and in an RA-treated hESC-CM. (**B**) Current–voltage (I–V) relationships of L-type Ca^2+^ current (I_Ca,L_) in Std and RA-treated hESC-CMs. (**C**) I–V relationships of steady-state currents in Std and RA-treated hESC-CMs. (**D**) I–V relationships of depolarization-activated steady-state currents in Std and RA-treated hESC-CMs in absence and presence of 50 µmol/L 4-aminopyridine (4-AP) to block ultrarapid delayed rectifier K^+^ current (I_Kur_), obtained by re-analysis of data obtained in the study by Devalla et al. [49]. (**E**) Voltage clamp protocol (top panel) and typical transient outward K^+^ current (I_to1_) recorded at +40 mV in a Std and in an RA-treated hESC-CM. (**F**) I–V relationships of I_to1_ in Std and RA-treated hESC-CMs. (**G**) I–V relationships of I_to1_ normalized to its largest amplitude, resulting in overlapping Std and RA curves, indicating that the voltage-dependence of I_to1_ activation is similar in Std and RA-treated hESC-CMs (*p* = 0.78 (two-way RM ANOVA)). (**H**) I–V relationships of voltage-dependence of I_to1_ inactivation (*p* = 0.99; two-way RM ANOVA). Solid lines: Boltzmann fits to mean data. Inset: Two-pulse voltage clamp protocol used. * *p* < 0.05, RA-treated vs. Std (two-way RM ANOVA).

In a subset of 13 Std and 14 RA-treated intrinsically quiescent hESC-CMs, the AP measurements were followed by voltage clamp experiments to examine net membrane currents. First, we measured the net currents in response to 500 ms hyperpolarizing and depolarizing voltage steps from a holding potential of −50 mV (Figure 2A, inset). Figure 2A shows typical membrane current recordings in response to voltage clamp steps to −100, 0, and 50 mV in a Std and in an RA-treated hESC-CM. The step to 0 mV activates an inwardly directed current, which we defined as I_Ca,L_ and which was significantly smaller in RA-treated hESC-CMs (Figure 2B). We defined the quasi-steady-state current during hyperpolarizing voltage steps as I_K1_, and its current density did not differ between Std and RA-treated hESC-CMs (Figure 2C). The quasi-steady-state current reverses at −68.0 ± 1.6 and −67.9 ± 2.1 mV (*p* = 0.96; *t*-test) for Std and RA-treated hESC-CMs, respectively, which is consistent with their MDP values (−70.1 ± 1.5 (Std) and −70.4 ± 1.4 (RA) mV; *p* = 0.86; *t*-test) and the strong relationship between the I_K1_ reversal potential and MDP [101]. The steady-state currents during the depolarizing steps are a mixture of several currents, including at least I_Kr_, the slow delayed rectifier K^+^ current (I_Ks_), and I_Kur_. The total current amplitude was significantly larger in RA-treated hESC-CMs (Figure 2C), likely due to the larger I_Kur_ in these cells [49]. We have now re-analyzed the experimental data from the same type of hESC-CMs that we had obtained in the study of Devalla et al. [49], and it has become evident that blockade of I_Kur_ with 50 μmol/L 4-aminopyridine (4-AP) abolished the initial differences in I_K_ (Figure 2D). These data, supported by the fact that I_Kr_ is not significantly different between Std and RA-treated hiPSC-CMs [34,68], demonstrate that I_Kur_ is responsible for the larger net steady-state currents during the depolarizing steps from a holding potential of −50 mV.

The membrane current measurements from a holding potential of −50 mV were followed by measurements with a holding potential of −80 mV (Figure 2E, inset) in the presence of CdCl_2_ to determine the transient outward K^+^ current, I_to1_ (see Section 2.2.3). Typical I_to1_ recordings upon depolarizing steps to +40 mV are shown in Figure 2E. The magnitude of I_to1_ differs between Std and RA-treated hESC-CMs, with a larger density in RA-treated hESC-CMs (Figure 2F). Neither the voltage dependence of activation (Figure 2G) nor the voltage dependence of inactivation (Figure 2H) differs between Std and RA-treated hESC-CMs (*p* = 0.78 (activation), *p* = 0.99 (inactivation); two-way RM ANOVA). Thus, the RA-treated hESC-CMs have larger repolarizing steady-state and transient K^+^ currents at positive potentials, while their depolarizing I_Ca,L_ is smaller, which well explains their faster repolarization and shorter APDs.

#### 3.1.3. AP Waveforms Related to Individual Membrane Currents

**Figure 3:**
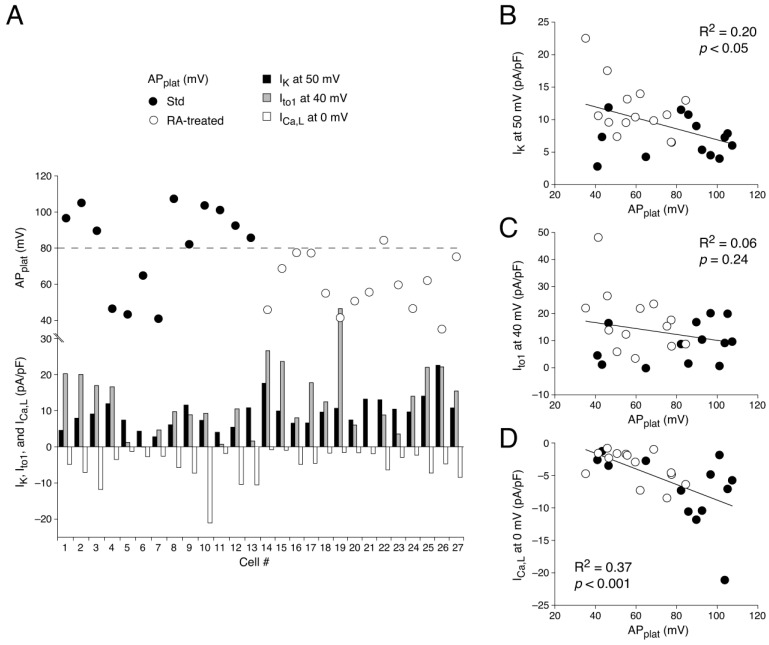
Relationships between AP_plat_ of hESC-CMs and their individual membrane currents. (**A**) Unbiased plot of AP_plat_ (open and filled circles, top), and I_K_, I_to1_, and I_Ca,L_ densities (open and filled bars, bottom) for all 27 cells measured. Dashed line indicates AP_plat_ of 80 mV. (**B**–**D**) Relationships between AP_plat_ and (**B**) I_K_, (**C**) I_to1_, and (**D**) I_Ca,L_ densities of all hESC-CMs studied. Solid lines: linear correlation fits (Pearson correlation tests), with R^2^ and *p* values as indicated.

Next, we related the individual membrane currents to the AP shape for each cell to discover the reason for the AP heterogeneity in the Std and RA-treated hESC-CMs. First, we plotted the measured AP_plat_ and I_K_, I_to1_, and I_Ca,L_ densities for all 27 cells in one figure (Figure 3A) for an unbiased presentation of the available data. It is immediately apparent from Figure 3A that there is a large scatter of values, consistent with the heterogeneity found in APs, but it is difficult to resolve correlations between AP_plat_ and individual membrane currents. Therefore, we next plotted each of the I_K_, I_to1_, and I_Ca,L_ densities against AP_plat_ (Figure 3B–D). The linear correlation fits demonstrate a moderate correlation between AP_plat_ and I_K_ (Figure 3B) and a strong correlation between AP_plat_ and I_Ca,L_ (Figure 3D), in the absence of a clear-cut correlation between AP_plat_ and I_to1_ (Figure 3C). This is somewhat surprising because several studies have concluded that both I_to1_ and I_K_, and especially I_Kur_, are important determinants of the plateau amplitude in stem cell-derived cardiomyocytes (see Section 4).

### 3.2. Influx and Efflux of Ca^2+^ Contribute to Differences in AP Shape

In Section 3.1, we demonstrated that I_Ca,L_, measured as the net inward current, is significantly lower in RA-treated hESC-CMs than in Std hESC-CMs, and that this current has the strongest relationship with the AP_plat_, and thus with an A-like AP shape. The measurements in Section 3.1 allow a direct correlation between the AP shape and individual ionic current densities in a cell, but it may be that the net inward current, which is largely carried by I_Ca,L_ during the AP plateau, is influenced by a number of outward currents that may be active at the same time during the AP and are substantially larger in RA-treated hESC-CMs (Figure 2). Therefore, we next determined the I_Ca,L_ characteristics in more detail in a separate set of experiments, using modified solutions and standard square-step voltage clamp protocols, as presented in Section 3.2.1. Because the AP shape also strongly determines the behavior of I_Ca,L_ [102,103,104], these square-step measurements were followed by AP clamp measurements using pre-recorded typical V- and A-like AP shapes (see Section 3.2.2). While I_Ca,L_ is important for Ca^2+^ influx, the Na^+^-Ca^2+^ exchanger is important for Ca^2+^ efflux [105]. Therefore, we also tested whether the current generated by the Na^+^-Ca^2+^ exchanger (I_NCX_) was different between Std and RA-treated hESC-CMs (see Section 3.2.3).

#### 3.2.1. I_Ca,L_ Determined by Square-Step Voltage Clamp Protocols

**Figure 4:**
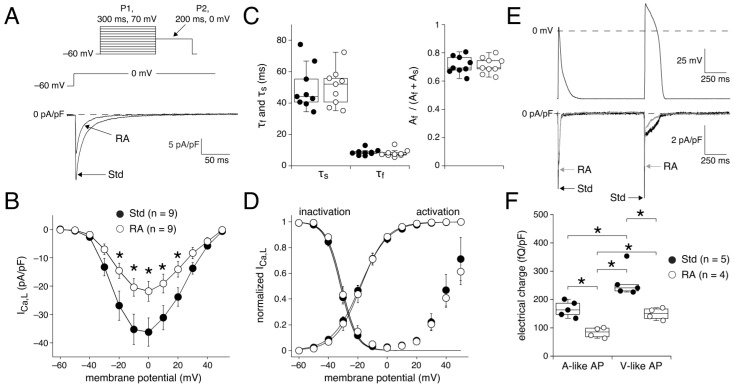
Characteristics of L-type Ca^2+^ current (I_Ca,L_) in Std and in RA-treated hESC-CMs analyzed with ruptured patch clamp. (**A**) Voltage clamp protocol (top panel) and typical I_Ca,L_ recorded at 0 mV in a Std and in an RA-treated hESC-CM. (**B**) I–V relationships of I_Ca,L_ density in Std and RA-treated hESC-CMs (* *p* < 0.05; two-way RM ANOVA followed by Students–Newman–Keuls post hoc test). (**C**) Associated box plots and individual data points of rate of I_Ca,L_ inactivation in response to depolarizing pulses from −60 to 0 mV (with fast and slow time constants τ_f_ and τ_s_, respectively; left) and relative amplitude of its fast and slow components (right). (**D**) Associated voltage-dependence of I_Ca,L_ activation (*p* = 0.90; two-way RM ANOVA) and voltage-dependence of I_Ca,L_ inactivation (*p* = 0.88; two-way RM ANOVA). Solid lines: Boltzmann fits to the mean data. (**E**) Top panel: AP clamp protocol consisting of pre-recorded A- and V-like APs from hESC-CMs. Bottom panel: typical example of I_Ca,L_ during the two AP shapes in a Std (black line) and in an RA-treated (gray line) hESC-CM. Arrows indicate the peak I_Ca,L_. (**F**) Box plots and individual data points of electrical charge carried by I_Ca,L_ during A- and V-like APs calculated from the area under associated I_Ca,L_ traces in Std and RA-treated hESC-CMs. * *p* < 0.05; two-way ANOVA followed by Students–Newman–Keuls post hoc test.

Figure 4A shows typical I_Ca,L_ traces in response to a depolarizing step from −60 to 0 mV in a Std and in an RA-treated hESC-CM. Mean I–V relationships are shown in Figure 4B and demonstrate that I_Ca,L_ density is significantly larger in Std compared to RA-treated hESC-CMs. For example at 0 mV, the average I_Ca,L_ was −36.2 ± 5.0 and −21.8 ± 3.5 pA/pF in Std and RA-treated hESC-CMs, respectively, indicating a 67% larger I_Ca,L_ density in Std hESC-CMs. The rate of I_Ca,L_ inactivation is similar in Std and RA-treated hESC-CMs at 0 mV, since their τ_f_, τ_s_, and relative amplitude of the fast and slow components of the current inactivation are not significantly different (Figure 4C; *p* = 0.63, 0.93, and 0.89 (*t*-test) for τ_f_, τ_s_, and relative amplitude, respectively). The voltage dependence of I_Ca,L_ activation and inactivation are shown in Figure 4D. Neither the voltage dependence of activation nor that of inactivation differs between Std and RA-treated hESC-CMs, as indicated by the virtually overlapping I–V relationships (*p* = 0.90 (activation), *p* = 0.88 (inactivation); two-way RM ANOVA). Typical for I_Ca,L_, the steady-state inactivation curve rises at membrane potentials positive to 0 mV (Figure 4D), due to voltage and Ca^2+^-dependent facilitation [106]. However, this process was not significantly different between the Std and RA-treated hESC-CMs.

#### 3.2.2. I_Ca,L_ Determined by AP Clamp Protocols

In general, standard square-step voltage clamp protocols are useful to determine bio-physical parameters in detail, but the functional effects of a membrane current are also importantly determined by the AP shape, as shown for example for I_Ca,L_ by Yuan et al. [102]. To assess the effect of AP shape on the total I_Ca,L_-mediated Ca^2+^ influx we digitized a typical V-like AP from a Std hESC-CM and a typical A-like AP from an RA-treated hESC-CM and combined them within the same AP clamp protocol trace (Figure 4E, top panel). Both Std and RA-treated hESC-CMs were exposed to the two repeating AP shapes and I_Ca,L_ was measured as the nifedipine-sensitive current. Typical recordings of I_Ca,L_ are shown in Figure 4E, bottom panel. During the plateau of the V-like AP, there is still a considerable activity of I_Ca,L_. Consequently, the electrical charge carried by Ca^2+^ ions entering the cell during a V-like AP is significantly larger than during an A-like AP in both Std and RA-treated hESC-CMs (Figure 4F). In addition, the electrical charge carried by Ca^2+^ ions entering the cell during both A- and V-like APs is significantly higher in Std hESC-CMs, likely due to the larger I_Ca,L_ densities in this cell type. Interestingly, the electrical charge through L-type Ca^2+^ channels is 215% larger (*p* < 0.05; two-way ANOVA) during a V-like AP cycle in Std hESC-CMs (265 ± 31 pQ/pF; Figure 4F, right set of closed symbols) than during an A-like AP cycle in RA-treated hESC-CMs (84 ± 10 pQ/pF; Figure 4F, left set of open symbols). Thus, our AP clamp experiments demonstrate that the difference in Ca^2+^ influx through I_Ca,L_ between V- and A-like APs is much more pronounced than analyzed from conventional square-step voltage clamp measurements, which revealed a 67% larger I_Ca,L_ density in Std hESC-CMs compared to RA-treated hESC-CMs (see Section 3.2.1).

#### 3.2.3. I_NCX_ Densities in Std and RA-Treated hESC-CMs

**Figure 5:**
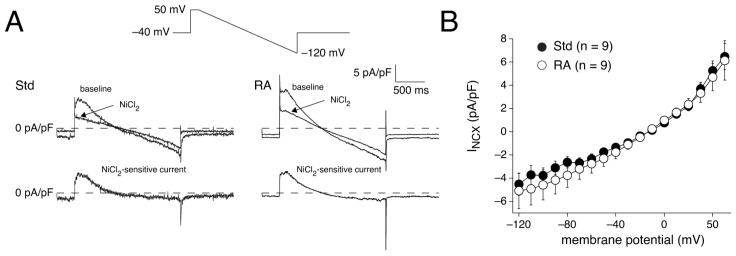
Na^+^-Ca^2+^ exchange current (I_NCX_) in Std and RA-treated hESC-CMs. (**A**) Typical traces of membrane current under baseline conditions and in the presence of 10 mmol/L NiCl_2_ in a Std (**left**) and in an RA-treated hESC-CM (**right**) measured during a descending ramp protocol (inset). I_NCX_ is obtained by subtraction as current sensitive to 10 mmol/L NiCl_2_ (bottom traces). (**B**) Average I–V relationships of I_NCX_.

The influx of Ca^2+^ through I_Ca,L_ must be extruded by the Na^+^-Ca^2+^ exchanger [105]. Therefore, we next measured the density of the Na^+^-Ca^2+^ exchange current (I_NCX_) in Std and RA-treated hESC-CMs using a pipette solution with a free Ca^2+^ concentration buffered at 150 nmol/L and a bath solution containing nifedipine to block I_Ca,L_. Thus, the recorded I_NCX_ is a measure of current density that is independent of the differences in Ca^2+^ influx through I_Ca,L_ between Std and RA-treated hESC-CMs, as determined in Section 3.2.2. Figure 5A shows typical membrane current recordings measured under baseline conditions and in the presence of NiCl_2_ during a descending ramp (inset). I_NCX_ was defined as the NiCl_2_-sensitive current. The I_NCX_ density did not differ significantly between Std and RA-treated hESC-CMs (*p* = 0.27; two-way RM ANOVA; Figure 5B).

### 3.3. Ca^2+^ Transients Reflect Ionic Current Differences During A- and V-like AP Shapes

**Figure 6:**
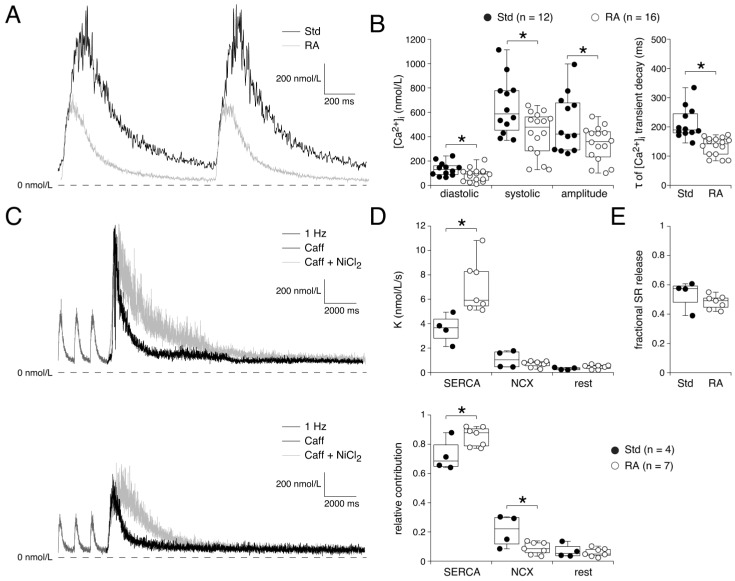
Intracellular Ca^2+^ homeostasis in Std and RA-treated hESC-CMs. (**A**) Typical intracellular Ca^2+^ concentration ([Ca^2+^]_i_) transients in a Std and in an RA-treated hESC-CM at 1 Hz pacing. (**B**) Left: Box plots and individual data points of diastolic and systolic [Ca^2+^]_i_ levels and associated [Ca^2+^]_i_ transient amplitude. Right: Box plots and individual data points of the time constant (τ) of the [Ca^2+^]_i_ transient decay. (**C**) Typical [Ca^2+^]_i_ transients in a Std (top) and in an RA-treated hESC-CM (bottom) at 1 Hz pacing and in response to a consecutive caffeine (Caff) pulse and in response to a consecutive Caff + NiCl_2_ pulse. (**D**) Box plots and individual data points of SERCA, NCX, and residual activity (rate constants K_SERCA_, K_NCX_, and K_rest_) for Ca^2+^ extrusion (top) and their relative contributions (bottom). (**E**) Box plots and individual data points of fraction of Ca^2+^ released from SR. * *p* < 0.05; RA-treated vs. Std (unpaired *t*-test or Mann–Whitney rank-sum test).

The above experiments demonstrate substantial differences in Ca^2+^ influx between Std and RA-treated hESC-CMs, which may have a major impact on the homeostasis of the intracellular Ca^2+^ concentration ([Ca^2+^]_i_). In a final series of experiments, we compared the [Ca^2+^]_i_ handling between Std and RA-treated hESC-CMs paced at 1 Hz. Figure 6A shows typical [Ca^2+^]_i_ transients from a Std and an RA-treated hESC-CM. Both systolic and diastolic [Ca^2+^]_i_ are lower in the RA-treated hESC-CM, as reflected in the average data in Figure 6B (left). The average [Ca^2+^]_i_ transient amplitude is also significantly smaller in RA-treated hESC-CMs (Figure 6B, left). The initial rise of the [Ca^2+^]_i_ transient is overlapping in the typical examples of Figure 6A, as reflected by the time it takes to reach a 100 nmol/L rise in [Ca^2+^]_i_. On average, the 100 nmol/L rise in [Ca^2+^]_i_ is reached after 16.1 ± 3.2 and 18.1 ± 4.8 ms in Std and RA-treated hESC-CMs, respectively (*p* = 0.37; Mann–Whitney rank-sum test). In contrast, the decay of the [Ca^2+^]_i_ transient is significantly faster in the RA-treated hESC-CMs, as reflected by the lower decay time constant obtained from mono-exponential fits to the decay phase of the [Ca^2+^]_i_ transients (Figure 6B, right).

The mechanisms underlying the faster decay of the [Ca^2+^]_i_ transient were further assessed by measurements of the activity of the three main cytoplasmic Ca^2+^ removal pathways [105,107,108]: (1) reuptake of Ca^2+^ into the SR by SERCA, (2) Ca^2+^ efflux through the Na^+^/Ca^2+^ exchanger, and (3) the slow Ca^2+^ uptake systems (mitochondrial Ca^2+^ uniporter and sarcolemmal Ca^2+^ ATPase). The SERCA activity (K_SERCA_) was analyzed by comparing the rates of decay of the 1 Hz triggered [Ca^2+^]_i_ transient and the caffeine-induced [Ca^2+^]_i_ transient (K_caff_) of the same cell (Figure 6C). Similarly, the NCX activity (K_NCX_) was obtained by subtracting K_caff_ from that of the caffeine-induced [Ca^2+^]_i_ transient in the presence of 10 mmol/L NiCl_2_ (K_rest_) (Figure 6C). K_SERCA_ is significantly higher in RA-treated hESC-CMs than in Std hESC-CMs, with no significant difference in K_NCX_ or K_rest_ (Figure 6D, top left; *p* = 0.79 (K_NCX_) and *p* = 0.53 (K_rest_), Mann–Whitney rank-sum test). The relative contribution of SERCA to the [Ca^2+^]_i_ transient decay is higher, whereas the relative contribution of NCX is lower, in RA-treated hESC-CMs than in Std hESC-CMs (Figure 6D, bottom). The relative contribution of the mitochondrial Ca^2+^ uptake together with the sarcolemmal Ca^2+^-ATPase does not differ between Std and RA-treated hESC-CMs (Figure 6D, bottom, rightmost bars; *p* = 0.24, *t*-test). The fraction of Ca^2+^ that is released from the SR during an AP was assessed using the ratio of the amplitude of the 1 Hz stimulated [Ca^2+^]_i_ transient to the amplitude of the 10 mmol/L caffeine-induced [Ca^2+^]_i_ transient as a measure of SR Ca^2+^ content. This fractional Ca^2+^ release was not different between Std and RA-treated hESC-CMs (Figure 6E; *p* = 0.66, *t*-test). The amplitude of the caffeine-induced [Ca^2+^]_i_ transient appeared to be smaller in RA-treated hESC-CMs (496 ± 102 (RA-treated) vs. 738 ± 86 (Std) nmol/L), but this difference did not reach the level of significance (*p* = 0.11, *t*-test), likely due to the relatively small number of cells.

## 4. Discussion

### 4.1. Overview

We found mixed populations of AP shapes in both the Std and RA-treated hESC-CMs. Using AP_plat_ as our criterion to separate cells with A-like and V-like APs, we demonstrated that 81% of the Std hESC-CMs but only 8% of the RA-treated hESC-CMs had V-like APs (Figure 1). Voltage clamp measurements revealed that hESC-CMs with A-like APs have a larger I_K_ (due to the atrial-specific I_Kur_) and I_to1_, as well as a smaller I_Ca,L_, than hESC-CMs with V-like APs (Figure 2, Figure 3 and Figure 4). Linear correlation fits of the current densities against the AP_plat_ demonstrate a strong correlation of AP_plat_ with I_Ca,L_ and a moderate correlation with I_K_, but no clear-cut correlation with I_to1_. Thus, apart from I_K_ (through I_Kur_), I_Ca,L_ also contributes to the different AP shapes. In addition, we observed substantial differences in [Ca^2+^]_i_ handling between Std and RA-treated hESC-CMs, in the absence of a difference in I_NCX_ density (Figure 5 and Figure 6). The Ca^2+^ influx during an AP cycle is 3.15 times higher in V-like hESC-CMs than in A-like hESC-CMs. This is not only due to the lower current density of I_Ca,L_ in A-like CMs, but also to the differences in the membrane potential course of the A- and V-like APs.

### 4.2. RA Treatment to Increase the Amount of CMs with an Atrial Fate

We used 1 μmol/L RA to induce differentiation towards atrial CMs, which together with 0.5 μmol/L RA is a common concentration used to generate A-like CMs. These concentrations result in the highest expression of the atrial-specific *KCNA5* gene [61,76] and the highest density of its associated membrane current, I_Kur_ [99]. In addition, it induces the expression of the atrial-specific *KCNJ3* and *KCNJ5* genes [49,61,72] and the associated acetylcholine-activated K^+^ current (I_K,ACh_) [45,49,74,94,98,109], which is virtually absent in CMs of Std differentiations. Even at this optimal concentration of RA, AP heterogeneity is commonly observed in patch clamp studies using single RA-treated CMs, as shown in Table 1 and our Figure 1. Using sharp electrode measurements, AP heterogeneity may be reduced in multicellular preparations [99], since this represents the average AP morphology of many cells due to electrotonic coupling [110]. AP heterogeneity is also observed in human atria, at least due to regional differences (see Elliott et al. [110] and primary references cited therein), but it remains to be elucidated whether this is due to a mixture of ‘regional’ cells within the atria or simply a cell-to-cell variation in membrane current densities. In any case, our study shows that the AP_plat_ of hESC-CMs shows clear correlations with APA and APD_20_, APD_50_, and APD_90_, suggesting that the AP heterogeneity is not due to the presence of purely ventricular or purely atrial CMs. This is further supported by the clear correlations between AP_plat_ and some membrane current densities, in particular those of I_K_ and I_Ca,L_.

In the present study, we used two different criteria based on AP shape to distinguish between V- and A-like hESC-CMs, i.e., APD_90/50_ < 2 and AP_plat_ > 80 mV. Both criteria gave the same percentage of V- and A-like cells in the Std hESC-CMs, but differed in their estimation of the percentage of V-like cells in the RA-treated cells (see Section 3.1.1). Using the APD_90/50_ < 2 criterion, 35% of the RA-treated hESC-CMs would be defined as V-like, while using AP_plat_ > 80 mV, only 8% of the RA-treated cells would be defined as V-like. Schwach et al. [61] used a dual atrial NKX2.5^eGFP/+^-COUP-TFII^mCherry/+^ reporter stem cell line to identify A-like hESC-CMs following cardiac differentiation and using this line 83.6% of Std hESC-CMs were V-like, while in RA-treated hESC-CMs this was 8.9%. These percentages are very close to what we observed using the AP_plat_ with a cut-off of 80 mV. In the current study, transcriptional phenotyping of single cells was not performed. Biendarra-Tiegs et al. [67] performed such experiments but were unable to link subtype-associated gene expression to subtype-specific electrophysiology. This could be related to the APD_90_/APD_50_ ratio of 1.4 that they used to separate atrial-like and ventricular-like hiPSC-CMs, but might also be related to the intrinsic heterogeneity between cells, even within subtype-specific cells, as demonstrated in our Figure 1 and Figure 3.

Although the AP_plat_ cut-off of 80 mV provided a relatively good distinction between A- and V-like hESC-CMs in the present study (Figure 1C), this cut-off value may require some minor adjustment when using other cell lines, differentiation protocols, RA-treatment, and/or measuring conditions, since average AP_plat_ values may differ between studies. For example, AP_plat_ values at 1 Hz pacing rate were significantly higher when dynamic clamp was used to provide these cells with a regular I_K1_ than when measurements were performed without dynamic clamp [75,96], likely due to the more negative MDP in the presence of a regular I_K1_, resulting in higher AP_plat_ values. Therefore, we used a cut-off value of 85 mV in a hiPSC-CMs study in which APs of wild-type and Brugada syndrome patient lines were measured using such dynamic clamp [111]. Additionally, AP_plat_ increases upon I_Kur_ knock-out [76] and I_Kur_ blockade [49,62,112] in RA-treated cells by reducing the repolarization rate. Therefore, we advise researchers to make their own estimate of the AP_plat_ cut-off value if the percentage of V- and A-like cells needs to be determined.

### 4.3. A- and V-like APs Explained by Membrane Current Differences

#### 4.3.1. MDP and I_K1_

We found that the MDP was similar in Std and RA-treated hESC-CMs. This finding is consistent with the results of many studies using Std and RA-treated hESC-CMs and hiPSC-CMs [31,45,48,61,76,95,97,109], but contrasts with the findings of Chapotte-Baldacci et al. [35] in hiPSC-CMs and with findings in native human atrial and ventricular cardiomyocytes [113,114,115]. In CMs, I_K1_ is important for setting the MDP and since it is smaller in native human atrial CMs [116,117], their MDP is less negative compared to native human ventricular CMs. Why hESC-CMs and hiPSC-CMs generated with Std or RA-treatment protocols in general do not show such differences may be due to the small or virtually absent I_K1_ in hESC-CMs and hiPSC-CMs [84], resulting in depolarized and/or spontaneously active CMs in Std as well as in RA-treated conditions.

#### 4.3.2. dV/dt_max_ and I_Na_

We found that dV/dt_max_ was higher in paced hESC-CMs compared to spontaneously active hESC-CMs (Table 2). This is a consistent finding in stem cell-derived cardiomyocyte research and is attributed to the more depolarized state and/or to the less negative take-off potential of the spontaneously active cells, resulting in more pronounced inactivation of Na^+^ channels (see Verkerk and Wilders [84] and primary references cited therein).

At 1 Hz pacing, dV/dt_max_ was not significantly different between Std and RA-treated hESC-CMs (Table 2), which is consistent with many other studies [31,34,45,49,61,62,97]. However, it should be noted that several studies have found a higher dV/dt_max_ in V-like cells [35,95,109], similar to that found in native human atrial and ventricular CMs [114]. For example, Goldfracht et al. [95] reported a dV/dt_max_ of 11.8 ± 1.7 and 6.8 ± 0.8 V/s in V- and A-like APs of hESC-CMs, respectively, but this difference is likely due to differences in I_Ca,L_ rather than in I_Na_, because I_Na_ channels will be largely inactivated at their RMP potentials (−60 mV) mentioned [84]. However, in the case of a more negative RMP, such as in the study of Seibertz et al. [109] on hiPSC-CMs, the difference in dV/dt_max_ might be due to the larger I_Na_ in V-like hiPSC-CMs compared to A-like hiPSC-CMs [35,109], although this is not a consistent finding [34]. The less negative V_1/2_ of inactivation in A-like hiPSC-CMs [35], counteracting the lower current density in these cells, may also explain why many studies do not observe differences in dV/dt_max_ between A- and V-like APs. Of note, we did not observe such less negative V_1/2_ of inactivation in the A-like hESC-CMs of the present study (Figure 4D).

#### 4.3.3. AP Repolarization and I_Kur_

The shape of the A- and V-like APs of our hESC-CMs differs widely, with lower APD_20_, APD_50_, APD_90_, and AP_plat_ in A-like cells, consistent with many other studies with Std and RA-treated hESC-CMs and hiPSC-CMs (see Section 3.1.1) and native human atrial and ventricular CMs [114,115,118]. Various membrane currents may contribute to these differences, as discussed below.

We found a larger I_K_ density in RA-treated hESC-CMs compared to Std hESC-CMs at positive potentials. As already mentioned in Section 3.1.2 and demonstrated in Figure 2D, this is due to the larger I_Kur_ in A-like compared to V-like hESC-CMs and hiPSC-CMs [35,49,62]. Accordingly, we observed a clear correlation between the larger I_K_ and the lower AP_plat_.

Kaplan et al. [119] strongly suggested that I_Kur_ is the major determinant of the A-like AP morphology, based on drug and dynamic clamp experiments. They demonstrated that APs of A-like hiPSC-CMs took on a V-like shape when treated with 50 μmol/L 4-AP to block I_Kur_. In addition, they observed that injection of a virtual I_Kur_ into V-like hiPSC-CMs, employing the dynamic clamp technique using oocytes expressing a cloned Kv1.5 current, resulted in APs similar to those of A-like hiPSC-CMs. Other studies also indicate that I_Kur_ is an important player in the fast phase-1 repolarization phase, but knock-out of *KCNA5* or I_Kur_ blockade by 4-AP in A-like hiPSC-CMs did not result in completely V-like APs [39,49,62,76,99,120], which is consistent with observations on native human atrial CMs (see Verkerk et al. [63], and primary references cited therein). This indicates that, although I_Kur_ plays an important role, differences in membrane currents other than I_Kur_ also contribute to the observed differences in AP parameters, in particular AP_plat_, as indeed found in the present study.

#### 4.3.4. AP Repolarization and I_to1_ Differences

We found a larger I_to1_ in RA-treated hESC-CMs compared to Std ones (Figure 2F). This is consistent with findings of Cordeiro et al. [46], who demonstrated that I_to1_ converted V-like APs into A-like APs in silico, to which end they turned the Luo-Rudy phase II guinea pig ventricular cell model [121] into a model of spontaneously active hiPSC-CMs by strongly reducing its I_K1_ current density. In addition, it is also consistent with the studies by Schulz et al. [99] and Chapotte-Baldacci et al. [35], who found that RA-treatment increased I_to1_ in hiPSC-CMs. A larger I_to1_ density in A-like hESC-CMs is also in line with native human CM studies, in which I_to1_ was found to be larger in freshly isolated atrial CMs than in ventricular CMs [113].

I_to1_ modulates the AP plateau and duration importantly [122]. Accordingly, the larger I_to1_ may contribute to the faster repolarization and smaller AP_plat_ in the RA-treated hESC-CMs. In this light, it is surprising that the correlation between the AP_plat_ and I_to1_ density in our study is relatively weak and not statistically significant (Figure 3C). This may, however, be due to the relatively depolarized membrane potential of our hESC-CMs, which results in voltage-dependent I_to1_ inactivation [96] and slow recovery from I_to1_ inactivation [46], both of which reduce the functional availability of this ion current. Therefore, it should not be concluded that the relatively weak correlation between the AP_plat_ and I_to1_ density demonstrates that I_to1_ does not play an important role in AP repolarization.

#### 4.3.5. AP Repolarization and I_Ca,L_ Differences

Using standard square-step voltage clamp protocols, we found a smaller I_Ca,L_ density in RA-treated hESC-CMs compared to Std ones, as determined by either the whole-cell ruptured patch clamp technique (Figure 4B) or the perforated patch clamp technique (Figure 2B). However, there is an apparent discrepancy in the I_Ca,L_ density between Figure 4B and Figure 2B. This discrepancy may be due to several reasons, as set out below.

First, the data in Figure 2B are from quiescent cells in Tyrode’s solution that were able to contract upon field stimulation, whereas the data in Figure 4B are from the entire population of cells, which is because the response to field stimulation could not be tested due to the non-physiological solutions optimized for ruptured patch I_Ca,L_ measurements. Quiescent cells are the minority in the overall cell population in the recording chamber, but are attractive for AP measurements, since their MDP is significantly more negative than that of spontaneously active cells (see also Table 2). Consequently, they have sodium current-driven APs [49,83]. In addition, any pacing frequency can be chosen without interference with spontaneous AP formation [49]. However, it is conceivable that the quiescent cells have a lower I_Ca,L_ density than the spontaneously active ones. Therefore, we have also analyzed voltage clamp data from spontaneously active Std hESC-CMs measured with the perforated patch clamp technique (and otherwise identical recording conditions as in Figure 2B). At 0 mV, their I_Ca,L_ density was −8.7 ± 1.9 pA/pF (*n* = 10), which is not significantly different from the I_Ca,L_ density in the quiescent Std hESC-CMs of Figure 2B (−6.3 ± 1.7 pA/pF, *n* = 13). Therefore, we exclude this as a potential source of the different I_Ca,L_ densities.

Second, there are essential differences in the recording conditions. The data in Figure 2B are from amphotericin-perforated patch clamp measurements with close-to-physiological solutions, whereas the data in Figure 4B are from ruptured patch clamp recordings with strongly modified pipette and bath solutions (including EGTA) optimized for selective I_Ca,L_ measurements. Ruptured patch clamp typically results in lower access resistances than perforated patch, and consequently voltage clamp control is better. In addition, it is known that EGTA (and other Ca^2+^ buffers, such as BAPTA) affects I_Ca,L_ density, with larger densities by higher Ca^2+^ buffer capacity [123,124]. We therefore attribute the higher I_Ca,L_ density in Figure 4B to the presence of EGTA and the use of the ruptured patch clamp technique.

Both hiPSC-CMs and hESC-CMs are responsive to β-adrenergic and muscarinic antagonists, with similar chronotropic effects in both cell types [64]. However, we did not test whether our hESC-CMs responded to β-adrenergic agonists such as isoproterenol to check on the possibility that the relatively high I_Ca,L_ density in Figure 4B represents a phosphorylated state of the I_Ca,L_ channels. Therefore, we cannot exclude the possibility that the relatively high I_Ca,L_ density in Figure 4B, in contrast to Figure 2B, is at least partly due to the I_Ca,L_ channels being in a phosphorylated state. Of note, we exclude the contribution of I_Na_ and the T-type Ca^2+^ current (I_Ca,T_) to our I_Ca,L_ measurements, since these currents are inactivated by our used voltage clamp protocol. Also, the measured voltage dependence of activation and inactivation, as well as the rate of current inactivation, are typical for I_Ca,L_.

The smaller I_Ca,L_ density in RA-treated hESC-CMs compared to Std ones of Figure 2B and Figure 4B is consistent with previous findings in hESC-CMs and/or hiPSC-CMs [34,35,45,68,109]. Such difference in I_Ca,L_ density has also been observed in native human atrial and ventricular CMs by Cohen and Lederer [118] (their Figure 4), Mewes and Ravens [125] (their Table 2), and Ouadid et al. [126] (their Table 1). Remarkably, the I_Ca,L_ densities in our study are up to ten times higher than those found in native CMs, in either case applying the whole-cell configuration of the patch clamp technique with EGTA in the recording pipette. Part of this difference can be explained by the difference in experimental temperature, now that the I_Ca,L_ density in ventricular myocytes exhibits a Q_10_ of 2.3 ± 0.6 (mean ± SD, *n* = 7) [127]. In our study, the experiments were performed at 36 ± 0.1 °C, whereas Cohen and Lederer [118], Mewes and Ravens [125], and Ouadid et al. [126] all performed their experiments at room temperature. Furthermore, it should be noted that there is quite some variation in the I_Ca,L_ densities that have been reported for native human cardiomyocytes as well as for hESC-CMs and/or hiPSC-CMs, even when the recording conditions are largely similar, making it difficult to compare I_Ca,L_ densities. However, a direct comparison was made in the study by Uzun et al. [128]. They found that the I_Ca,L_ density of hiPSC-CMs was ≈2-fold larger than that of native human CMs. The exact reason for the smaller I_Ca,L_ in native human CMs remains unknown, but we cannot exclude that the smaller I_Ca,L_ is due to the CMs’ origin from patients with heart failure who received β-blockers [128]. In addition, it may be related to the hiPSC-CM culturing methods, as these affect I_Ca,L_ densities [57,128]. Lastly, we exclude the immaturity of hiPSC-CMs because maturation increases I_Ca,L_ rather than decreasing it [65].

We observed no difference in half-maximal activation and inactivation potentials (V_1/2_) between A-like and V-like hESC-CMs (Figure 4D), in agreement with findings in hiPSC-CMs [35,68] and native human atrial and ventricular CMs [118]. However, it should be noted that this is not a completely consistent finding, since Argenziano et al. [45] found a rightward shift of (in)activation properties in RA-treated hiPSC-CMs, whereas both Mewes and Ravens [125] (their Table 2) and Ouadid et al. [126] (their Table 2) observed a leftward shift of the V_1/2_ of activation in native atrial CMs. The rate of I_Ca,L_ inactivation was not different between Std and RA-treated hESC-CMs (Figure 4C), consistent with previous findings in hiPSC-CMs [35] and freshly isolated human CMs [118,126].

Previously, expression of the *CACNA1C* (Ca_V_1.2) and *CACNA1D* (Ca_V_1.3) genes has been found in hESC-CMs and hiPSC-CMs, with a higher gene expression in RA-treated cells [29,35,77,97,129]. At present, no pharmacological tools exist that are suitable to confirm or refute a role for Ca_V_1.3 channels in cellular responses, as reviewed recently [130]. Ca_V_1.2 and Ca_V_1.3 have slightly different activation kinetics and Ca_V_1.3 is activated at a more negative membrane potential [131]. However, the I–V curves of I_Ca,L_ overlap in our A- and V-like hESC-CMs, suggesting that the contribution of Ca_V_1.3 to our total I_Ca,L_ is limited, consistent with the idea that Ca_V_1.2 is the predominant I_Ca,L_ channel in the working myocardium [106,132,133].

A finding from the present study that substantially adds to our current knowledge is that there is a longer and stronger activity of I_Ca,L_ during the plateau of V-like APs compared to A-like APs. Consequently, the electrical charge due to Ca^2+^ ions entering the cell during a V-like AP is significantly larger than during an A-like AP. Yuan et al. [102] had performed AP clamp experiments using rabbit and rat ventricular APs and they found a larger Ca^2+^ influx using the rabbit AP (with a positive plateau level) than the rat AP (with a very negative plateau level), which is thus highly similar to our observation using A- and V-like APs. The substantially smaller Ca^2+^ influx through I_Ca,L_ is thus an important cause of the shorter, A-like APs of the RA-treated hESC-CMs. This is supported by the strong correlation between AP_plat_ and I_Ca,L_ (Figure 3D) and further substantiated by drug experiments, where RA-treated cells do not respond with AP changes to I_Ca,L_ blockade, while their non-RA-treated V-like counterparts show a large AP shortening [34,97,134].

#### 4.3.6. AP Repolarization and I_NCX_

Under Ca^2+^-buffered conditions, we found that the I_NCX_ density did not differ between RA-treated and Std hESC-CMs (Figure 5), consistent with the similar absolute K values of the NCX during the [Ca^2+^]_i_ transient decay phase (Figure 6D, top). The relative contribution of NCX to the removal of Ca^2+^ from the cytosol (Figure 6D, bottom) is, however, smaller in RA-treated hESC-CMs because the SERCA is much faster in RA-treated hESC-CMs (Figure 6D, top left). Additionally, and maybe even more importantly, the influx of Ca^2+^ is much lower in RA-treated hESC-CMs (Figure 4), and therefore fewer Ca^2+^ ions need to be removed from the cytosol by the NCX [105] to maintain the well-known Ca^2+^ flux balance [108]. This will result in a smaller I_NCX_ [135]. Since the I_NCX_ is an inwardly directed, depolarizing current during the plateau and final repolarization phases of APs [136], it importantly regulates APD [105]. While not demonstrated in the present study, it is known from other studies that NCX blockade in stem cell-derived cardiomyocytes results in shorter APs, as demonstrated in silico using hiPSC-CM computer models [65,136] and in vitro using the selective NCX blocker SEA0400 on hiPSC-CM engineered heart tissue [136]. This suggests that a lower I_NCX_ plays an additional important role in the shorter APDs of the RA-treated hESC-CMs, although further studies are needed to confirm this. Since Ca^2+^ homeostasis is very important in AP modulation, with essential differences between A- and V-like APs, we advise to avoid Ca^2+^ buffering substances like EGTA or BAPTA in pipette solutions for AP measurements. This is especially relevant for the use of A-like hiPSC-CMs in models of atrial fibrillation, since I_NCX_ is upregulated in patients with atrial fibrillation [137].

### 4.4. [Ca^2+^]_i_ Transient Differences

In adult CMs of various species including humans, Ca^2+^ signaling shows marked differences between atria and ventricles, as reviewed in detail by Dobrev et al. [138], Bootman et al. [139], Greiser [140], and Denham et al. [141]. An important source for Ca^2+^ transient differences between adult atrial and ventricular CMs is related to the transverse (T) tubule system [142]. Adult ventricular CMs have well-developed T-tubuli, but these are less pronounced or nearly absent in adult atrial CMs, resulting in a slower and more heterogeneous Ca^2+^ transient within an atrial CM (for reviews, see Bootman et al. [139], Greiser [140], and Denham et al. [141]). T-tubuli are virtually absent in both Std and RA-treated hESC-CMs [95], but we still observed marked differences between the Std and RA-treated CMs.

We found a lower [Ca^2+^]_i_ transient amplitude in RA-treated hESC-CMs (Figure 6B), which is consistent with a previous study in hiPSC-CMs [45]. The amplitude of the [Ca^2+^]_i_ transient is modulated by a number of factors, including the magnitude of I_Ca,L_, SR Ca^2+^ content, fractional SR release, and diastolic Ca^2+^ levels (for reviews, see Guatimosim et al. [143], Neef and Maier [107], Eisner [108], and Eisner et al. [144]). Except for the fractional release from the SR, these parameters are all smaller, or show a tendency to be smaller, in RA-treated CMs, explaining the lower [Ca^2+^]_i_ transient amplitude in this CM type. The Ca^2+^ transient was shorter in duration due to a faster decay in the RA-treated hESC-CMs (Figure 6B), which is consistent with various Ca^2+^ transient studies in hiPSC-CMs [35,45,77,97]. The decay phase of the [Ca^2+^]_i_ transients was faster due to a higher SERCA activity rather than an increased Ca^2+^ efflux via a higher NCX activity (Figure 6D). In human hearts, atrial CMs have higher SERCA but lower phospholamban (PLB) protein levels [145], and consequently, an enhanced Ca^2+^ uptake by SERCA in atrial CMs is a consistent finding in human CMs [146], and also in adult CMs of various other species [147,148,149]. Higher SERCA activity is supposed to increase the SR Ca^2+^ content and Ca^2+^ transient amplitude (for reviews, see Bers [105], Neef and Maier [107], and Eisner [108]). However, this was not the case in our measurements, suggesting that potential effects on SR Ca^2+^ content are compensated by the decrease in Ca^2+^ influx during the short APD of A-like APs [140]. The diastolic [Ca^2+^]_i_ was lower in RA-treated CMs (Figure 6B). This is likely due to the enhanced SERCA reuptake [144] in combination with a lower Ca^2+^ influx through I_Ca,L_ [150]. Various studies on hiPSC-CMs have reported a faster rise time of the Ca^2+^ transient [77], resulting in an earlier time-to-peak of the Ca^2+^ transient in RA-treated cells [45,77,97]. Such conclusions are frequently based on non-ratiometric imaging of the Ca^2+^ transients, where the recordings are often normalized [144]. Our ratiometric Ca^2+^ transient analysis, however, demonstrates that the release is not faster, but the [Ca^2+^]_i_ transient amplitude is much lower, resulting in a peak earlier in time.

### 4.5. Spontaneous Beating Rate

We found a shorter cycle length in spontaneously beating RA-treated hESC-CMs compared to the Std hESC-CMs, consistent with many other studies [35,39,45,72,77,132]. Since pacing rate influences various AP parameters, we have mainly used intrinsically quiescent cells paced at 1 Hz for our study, but some studies on Std and RA-treated CMs indicate differences in genes and/or the associated currents that may contribute to (differences in) pacemaking. Lower expression of *KCNJ2* (encoding the pore-forming Kir2.1 subunit of the I_K1_ channel) but higher expression of *CACNA1H* (encoding the pore-forming Ca_V_3.2 subunit of the T-type Ca^2+^ channel) as well as *HCN4* and *HCN1* (encoding the pore-forming HCN4 and HCN1 subunits of the I_f_ channel) and/or the associated currents have been reported in RA-treated CMs [35,45,68,72,77,95]. This would favor faster pacemaking [151]. On the other hand, the expression of *CACNA1C* (encoding the pore-forming subunit of the Ca_V_1.2 I_Ca,L_ channel) and/or its associated I_Ca,L_ has been found to be higher in Std CMs ([45,68,97], present study) and I_Ca,L_ is also important for pacemaking [151], complicating a simple and straightforward explanation for the shorter cycle length in the RA-treated CMs. Apart from differences in pacemaking-modulating genes and currents, the AP is shorter in RA-treated CMs, further promoting a shorter cycle length. Further studies are required to elucidate the exact mechanisms involved in the faster pacemaking in RA-treated CMs.

### 4.6. Limitations

Although some studies have demonstrated the presence of Ca^2+^-activated currents, such as the Ca^2+^-activated Cl^−^ current (I_Cl(Ca)_) and small, intermediate, and large conductance Ca^2+^-activated K^+^ currents, in hiPSC-CMs [152,153], we have not specifically assessed the presence of these currents in our hESC-CMs. However, our specific I_Ca,L_ measurements were performed using the ruptured patch clamp methodology with EGTA in the recording pipette solution. Under these conditions, we observed neither Ca^2+^-activated K^+^ currents in our hESC-CMs nor any current typical of I_Cl(Ca)_ [154]. Also, Zhao et al. [152] performed AP measurements in the presence of EGTA. However, the AP shape and duration were unaffected by apamine, a selective Ca^2+^-activated K^+^ current blocker, indicating that this current is not functional under these conditions. Furthermore, we have measured I_Ca,L_ during the AP clamp measurements with EGTA in the pipette solution as current sensitive to 10 µmol/L nifedipine. At this concentration, nifedipine completely blocks I_Ca,L_ [88], but it may also reduce I_Ca,T_ by 14% [155], if present. However, the rate of I_Ca,T_ inactivation is very fast and consequently the total amount of Ca^2+^ flux via I_Ca,T_ is small compared to that via I_Ca,L_ and likely negligible in working CMs [156]. Therefore, we attribute the currents shown in Figure 4 to I_Ca,L_ rather than to other currents.

The type of cardiomyocyte (N-, A-, or V-like) under investigation was not established in the case of the ruptured patch clamp-measured I_Ca,L_ and I_NCX_, and in the [Ca^2+^]_i_ measurements. Although the majority of Std hESC-CMs belong to V-like cardiomyocytes and the majority of RA-treated hESCs-CMs belong to A-like cardiomyocytes (Figure 1), a part of the variability in the results may stem from differences between cardiomyocyte types. To address this issue in the future, simultaneous recording of APs and intracellular Ca^2+^ will help to distinguish cardiomyocyte type and additionally give temporally synchronized information on the interplay between [Ca^2+^]_i_ handling and APs. While simultaneous optical voltage and Ca^2+^ transient recordings are possible [77,97], distinguishing A- and V-like cardiomyocytes is not straightforward in the case of ruptured patch clamp using non-physiological solutions. Selection of a specific cell type could potentially be based on contractions, since A-like cardiomyocytes have faster and shorter contractions. In the past, we have selected beating cardiomyocytes in the same bath solution as used for APs, after which the bath solution was switched to specific extracellular solutions [111]. Thus, the type of contractions before the use of strongly modified solutions may be used as an indication of a particular cardiomyocyte type. Another option is to use specific markers for particular cell types. One might consider the level of expression of dyadic junctions (‘dyads’), which is generally lower in atrial cardiomyocytes than in ventricular ones [157,158]. However, this is not feasible as hESC-CMs lack the highly developed and organized T-tubular system found in adult ventricular myocytes [159,160,161]. Schwach et al. [61] sorted pure A-like cell populations based on the presence of chick ovalbumin upstream promoter transcription factors (COUP-TFs) that were fused with a fluorescent reporter gene (mCherry). Another option is to use modified differentiation methods [162] and/or the RA inhibitor BMS493 [34], which yields populations of pure V-like hiPSC-CMs.

Of note, our study was performed in hESC-CMs and not in hiPSC-CMs. However, the percentages of N-, A-, and V-like cardiomyocytes are largely similar in hiPSC- and hESC-derived cardiomyocytes [29,163]. In addition, hESC-CMs and hiPSC-CMs are functionally and transcriptionally highly similar [163,164,165,166]. This suggests that AP heterogeneity in hiPSC-CMs can also be explained by a heterogeneous distribution of ionic currents, but further experiments are needed to confirm this hypothesis.

We performed our current clamp measurements without the use of dynamic clamp, while we and others (see Verkerk and Wilders [84] and primary references cited therein) have previously strongly advocated its use for iPSC-CM experiments in order to provide these cells with a regular I_K1_. The reason is that we wanted to link the AP morphology and parameters to ionic currents without the interference of in vitro injected currents, which may affect the APD_90/50_ ratio, a frequently used marker to describe triangulation of APs (Table 1). However, to overcome the spontaneous activity, we selected intrinsically quiescent CMs that were able to contract upon field stimulation, as set out in Section 4.5 above.

## 5. Conclusions

Our study on hESC-CMs contributes to a better understanding of (differences between) A-like and V-like APs in stem cell-derived CMs and in particular highlights the importance of I_Ca,L_ and [Ca^2+^]_i_ homeostasis in the observed AP heterogeneity. Such information is indispensable for understanding the basic AP morphology [167] and drug effects [49,97], for the generation and improvement of hiPSC-CM computer models [168], and for the design and/or conception of monolayers [31], engineered composite heart tissue [72,169], and engineered heart tissue models of atrial fibrillation [95].

## Figures and Tables

**Table 1:** Various action potential criteria used to identify cardiac AP subtypes in control hiPSC-CMs and hESC-CMs.

Study	Diff.	Temp. (°C)	EGTA	Criterion for N-like	Criterion for A-like	Criterion for V-like	N-|A-|V-like (%)
Itzhaki et al. [26]	Std	32	+	N/A	N/A	N/A	13|27|60
Itzhaki et al. [27]	Std	32	+	N/A	N/A	N/A	14|30|56
Li et al. [28]	Std	Room	−	N/A	N/A	N/A	N/A|N/A|81
Streckfuss-Bömeke et al. [25]	Std	Room	−	N/A	N/A	N/A	29|17|54 ^†,A^ 20|20|60 ^†,B^ 29|19|52 ^†,C^
Lee et al. [29]	Std	N/A	N/A	N/A	N/A	N/A	45|17|38
Lan et al. [30]	Std	36–37	+	N/A	N/A	N/A	≈5|35|60
Laksman et al. [31]	Std RA	22–23	−	N/A	N/A	N/A	7|≈13|≈80 ≈7|≈87|≈7
De la Roche et al. [32]	Std	Room	+	N/A	20 ms < APD_50_ < 200 ms	APD_50_ > 200 ms	N/A|12|88 ^‡^
Peng et al. [33]	Std	35	−	Slow upstrokes and fast pacing rate	APD_90_ < 150 ms	APD_90_ > 150 ms	<1|18|82
Pei et al. [34]	Std RA	35	−	Slow upstrokes and fast pacing rate	APD_90_ < 150 ms	APD_90_ > 150 ms	N/A|N/A|93 N/A|90|N/A
Chapotte-Baldacci et al. [35]	Std RA	Room	+	APD_90_ < 250 ms, APD_50_ − APD_20_ ≤ 10 ms	APD_90_ < 250 ms, APD_50_ − APD_20_ > 10 ms	APD_90_ > 250 ms	0|31|69 ^#^ 9|66|27 ^#^
Jara-Avaca et al. [36]	Std	37	+	Bell-shaped APs, APD_90/50_ > 1.4	APD_50_ < 100 ms, APD_90/50_ > 3	APD_50_ > 100 ms, APD_90/50_ < 1.4	0|≈33|≈63
Bett et al. [37]	Std	Room	+	N/A	APD_30_ < 300 ms, APD_30/90_ < 0.75	APD_30_ > 300 ms, APD_30/90_ > 0.75	N/A|≈64|≈28 ^#^
Kim et al. [38]	Std	Room	+	N/A	APD_30_ < 300 ms, APD_30/90_ < 0.75	APD_30_ > 300 ms, APD_30/90_ > 0.75	<1|19|68
Altomare et al. [39]	Std RA	35	−	N/A	APD_20/90_ < 0.44	APD_20/90_ > 0.44	N/A|34|66 N/A|74|26
Moretti et al. [40]	Std	35	+	Depolarized; low dV/dt, low APA	1.3 < APD_90/50_ < 1.6	1.1 < APD_90/50_ <1.3	18|20|62
El-Battrawy et al. [41]	Std	36	+	1.4 < APD_90/50_ < 1.7	APD_90/50_ > 1.7	APD_90/50_ < 1.4	5|22|73
Matsa et al. [42]	Std	37	+	1.4 < APD_90/50_ < 1.7	APD_90/50_ > 1.7	APD_90/50_ < 1.4	N/A|N/A|N/A
Hayano et al. [43]	Std	36–37	+	APD_90/50_ > 1.2, APA < 60 mV	APD_90/50_ > 1.2, APA > 80 mV	APD_90/50_ ≤ 1.2, APA > 80 mV	14|38|48
Ma et al. [44]	Std	35–37	−	APD_30–40_/APD_70–80_ < 1.5, dV/dt_max_ < 10 V/s	APD_30–40_/APD_70–80_ < 1.5	APD_30–40_/APD_70–80_ > 1.5	22|24|54
Argenziano et al. [45] *	Std RA	N/A	−	APD_30–40_/APD_70–80_ < 1.5, dV/dt_max_ < 10 V/s	APD_30–40_/APD_70–80_ < 1.5	APD_30–40_/APD_70–80_ > 1.5	7|7|86 6|85|9
Cordeiro et al. [46] *	Std	37	−	N/A	APD_30–40_/APD_70–80_ < 1.5	APD_30–40_/APD_70–80_ > 1.5	N/A|54|46 ^D^
Zhang et al. [47]	Std	Room	+	APD_30–40_/APD_70–80_ < 1.5, dV/dt_max_ < 10 V/s	APD_30–40_/APD_70–80_ < 1.5	APD_30–40_/APD_70–80_ > 1.5	18|65|17 ^E^ 0|50|50 ^F^
Ma et al. [48]	Std	37	−	APD_30–40_/APD_70–80_ < 1.5, dV/dt_max_ < 10 V/s	APD_30–40_/APD_70–80_ < 1.5	APD_30–40_/APD_70–80_ > 1.5	11|15|74
Devalla et al. [49]	Std RA	36	−	N/A	AP_plat_ < 80 mV	AP_plat_ > 80 mV	<1|≈19|≈80 <1|≈85|≈14

Percentages of N-, A-, and V-like cells do not add up to 100% in some cases due to cells that did not meet any of the AP criteria; * sharp microelectrode recordings from monolayers or clusters; ^†^ excluding Purkinje-like hiPSC-CMs; ^‡^ excluding atypical hiPSC-CMs; ^A^ derived from mesenchymal stem cells; ^B^ derived from hair keratinocytes; ^C^ derived from fibroblasts; ^D^ recalculated based on their source (their Table 1); ^E^ early and ^F^ late stage hiPSC-CMs in 2D and 3D differentiated preparations; ^#^ using dynamic clamp. APA: AP amplitude; APD_20_, APD_30_, APD_50_, and APD_90_: AP duration at 20, 30, 50, and 90% of repolarization, respectively; APD_20/90_: ratio between APD_20_ and APD_90_; APD_30–40_: time difference between APD_30_ and APD_40_; APD_30/90_: ratio between APD_30_ and APD_90_; APD_70–80_: time difference between APD_70_ and APD_80_; APD_90/50_: ratio between APD_90_ and APD_50_; AP_plat_: AP plateau amplitude at 20 ms after AP upstroke; dV/dt_max_: maximum AP upstroke velocity.

**Table 2:** Action potential (AP) parameters in Std and RA-treated hESC-CMs.

	Spontaneously Active		Quiescent—1 Hz Stimulation	
	Std (*n* = 15)	RA-Treated (*n* = 10)	Std (*n* = 36)	RA-Treated (*n* = 37)
Cycle length (ms)	814 ± 69	591 ± 56 *	N/A	N/A
MDP (mV)	−63.7 ± 1.9	−62.0 ± 2.0	−69.4 ± 0.8 ^#^	−70.1 ± 0.8 ^#^
APA (mV)	90.1 ± 4.5	76.9 ± 3.7 *	98.2 ± 2.4	80.3 ± 2.2 *
AP_plat_ (mV)	87.5 ± 4.8	70.7 ± 5.6 *	89.9 ± 3.3	60.3 ± 2.8 *
dV/dt_max_ (V/s)	8.4 ± 1.2	7.9 ± 2.5	60.3 ± 11.3 ^#^	41.4 ± 6.8 ^#^
APD_20_ (ms)	107.1 ± 8.8	64.2 ± 13.6 *	74.9 ± 8.3 ^#^	26.8 ± 3.9 *^,#^
APD_50_ (ms)	191.1 ± 21.5	103.9 ± 20.3 *	121.5 ± 12.6 ^#^	59.4 ± 7.1 *
APD_90_ (ms)	267.7 ± 30.1	162.6 ± 21.2 *	185.8 ± 8.5 ^#^	152.8 ± 8.5

MDP: maximum diastolic potential; APA: AP amplitude; AP_plat_: AP plateau amplitude at 20 ms after the AP upstroke; dV/dt_max_: maximum AP upstroke velocity; APD_20_, APD_50_, and APD_90_: AP duration at 20, 50, and 90% of repolarization, respectively. * *p* < 0.05, RA-treated vs. Std; ^#^ *p* < 0.05, spontaneously active vs. intrinsically quiescent upon 1 Hz stimulation (two-way ANOVA followed by Students–Newman–Keuls post hoc test).

## Data Availability

The raw data supporting the conclusions of this article will be made available by the authors on request.
